# ZNF191 alters DNA methylation and activates the PI3K‐AKT pathway in hepatoma cells via transcriptional regulation of *DNMT1*

**DOI:** 10.1002/cam4.4535

**Published:** 2022-01-28

**Authors:** Yufeng Liu, Hanghang Cheng, Chenchen Cheng, Fengyun Zheng, Zhonghua Zhao, Qi Chen, Wenjiao Zeng, Pingzhao Zhang, Cheng Huang, Wei Jiang, Xiuping Liu, Guoyuan Liu

**Affiliations:** ^1^ Department of Pathology School of Basic Medical Sciences Fudan University Shanghai China; ^2^ Institutes of Biomedical Sciences Fudan University Shanghai China; ^3^ Department of Liver Surgery & Transplantation Liver Cancer Institute Zhongshan Hospital Fudan University Shanghai China; ^4^ Key Laboratory of Metabolism and Molecular Medicine The Ministry of Education Department of Biochemistry and Molecular Biology School of Basic Medical Sciences Fudan University Shanghai China; ^5^ Department of Pathology Shanghai Fifth People’s Hospital School of Basic Medical Sciences Fudan University Shanghai China

**Keywords:** DNA methylation, DNMT1, hepatocellular carcinoma, PI3K‐AKT signaling pathway, zinc finger transcription factor 191(ZNF191)

## Abstract

**Background:**

Alteration of DNA methylation is an important event in pathogenesis and progression of hepatocellular carcinoma (HCC). DNA methyltransferase (DNMT) 1, the foremost contributor in DNA methylation machinery, was revealed elevated in HCC and significantly correlates with poor prognosis. However, the transcriptional regulation of *DNMT1* in HCC remains unknown.

**Methods:**

Real‐time PCR and immunohistochemistry were performed to detect DNMT1 and zinc finger transcription factor 191 (ZNF191) expressions in HCCs. Transcription activity of *DNMT1*promoter was analyzed with Luciferase reporter activity assay. The binding capacity of ZNF191 protein to *DNMT1* promoter was examined with chromatin immunoprecipitation‐qPCR (ChIP‐qPCR) and electrophoretic mobility shift assay (EMSA). DNA methylation level of hepatoma cells was detected with Methylation array.

**Results:**

ZNF191 can regulate DNMT1 mRNA and protein expression positively, and increase the transcription activity of the *DNMT1* promoter. ChIP‐qPCR and EMSA revealed that ZNF191 protein directly binds to the *DNMT1* promoter at nt‐240 AT(TCAT)_3_TC. Moreover, DNMT1 and ZNF191 expression correlate positively in human HCCs. With methylation array, DNA methylation alteration was observed in hepatoma cells with ZNF191 knockdown, and the differential methylation sites are enriched in the PI3K‐AKT pathway. Furthermore, we proved DNMT1 contributes the effect of ZNF191 on hepatoma cell growth via the PI3K‐AKT pathway.

**Conclusion:**

ZNF191 is a novel transcription regulator for *DNMT1*, and the pro‐proliferation effect of ZNF191/DNMT1/p‐AKT axis in hepatoma cells implies that ZNF191 status in HCCs may affect the therapeutic effect of DNMTs inhibitors and PI3K inhibitors for precise treatment of the disease.

## Graphical abstract

ZNF191 directly binds to *DNMT1* promoter, and transactivates the *DNMT1* gene. ZNF191 alters DNA methylation in hepatoma cells with DMS enriched in the PI3K‐AKT pathway. DNMT1 contributes the effect of ZNF191 on HCC cell growth via the PI3K‐AKT pathway.

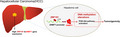

## 1 INTRODUCTION

Hepatocellular carcinoma (HCC) is a primary liver cancer and the fifth most common cancer worldwide, which represents the second leading cause of cancer‐related death worldwide.^1^ The development of HCC was reported to be driven by the interaction of virus infection (HBV and/or HCV) and various environmental factors (metabolic syndrome, alcohol, aflatoxin B1, and aristolochic acid) in genetically predisposed individuals^2^, ^3^ and the process is multistep and long‐term, which consisted of genetic and epigenetic alterations.^4^, ^5^

Alteration of DNA methylation is a common epigenetic abnormality identified in HCC,^6^, ^7^, ^8^ and probably involved in all stages of hepatocarcinogenesis through aberrant silencing the expression of tumor suppressor genes (TSG), or activating oncogenes with abnormal methylation of 5‐CpGs of target gene promoters, abnormal changes in chromatin structure, DNA conformation, and stability interaction, etc.^4^, ^8^ Identifying novel regulators of DNA methylation in HCCs may provide potential novel therapeutic targets for HCCs.

DNA methylation is catalyzed by DNA methyltransferases (DNMTs) in mammalian cells, including DNMT1, 3A, and 3B. Among them, DNMT1 is the most abundant and the main enzyme, which is responsible for de novo methylation to maintain genomic methylation patterns during replication.^9^ Aberrant high expression of DNMT1 mRNA and protein in human HCCs has been reported in different cohorts, and may play an important role during HCC development.^6^, ^10^, ^11^, ^12^, ^13^, ^14^ Thus transcription deregulation of the *DNMT1* gene may be an important factor during hepatocarcinogenesis, in addition to its post‐transcriptional auto‐inhibitory controls and posttranslational modifications, etc.^15^ Several transcription factors such as P53,^16^ SP1,^17^ E2F,^18^ AP1,^19^ and ERE1/2,^20^ have been identified to bind to the *DNMT1* promoter with high affinity, and be involved in distinct biological processes or tumorigenic conditions.^15^ However, the mechanism of transcriptional regulation of *DNMT1* gene in human HCCs is presently unknown.

The human zinc finger protein 191 (ZNF191, also called ZNF24), a Krüppel‐like protein,^21^ specifically interacts with the TCAT motif ex vivo,^22^ plays important roles in HCC development and progression.^23^, ^24^, ^25^ In this study, we revealed for the first time ZNF191 can bind to the *DNMT1* promoter directly, transactivate the *DNMT1* gene, induce DNMT1 mRNA and protein expression, and cause DNA methylation alteration in hepatoma cells, which may promote hepatoma cell proliferation by activating the PI3K‐AKT pathway. The findings suggest a possible role of ZNF191 in association with regulating epigenetic alterations in hepatocarcinogenesis.

## 2 MATERIALS AND METHODS

### 2.1 Tumor samples and tissue microarrays (TMAs)

Fresh surgical tissues of human HCCs (*n* = 44) and TMAs containing of paired (*n* = 149) HCC and adjacent nontumor tissues were obtained as described in our previous study.^25^ Informed consent was collected from each subject or subject's guardian after approval by Zhongshan Hospital Ethics Committee, Fudan University. The mean age of the patients was 52.3 years (range, 22–77 years). The last follow‐up was June 2014, with a median follow‐up of 23.53 months (range, 0.5–76 months).

### 2.2 Luciferase reporter assays

The wild type or mutant type of reporter construct (a 1074‐bp section of the *DNMT1* promoter cloned into the pGL3 luciferase plasmid) was co‐transfected into HEK‐293T cells with pCMV‐ZNF191‐FU (full length) or the negative control pCMV‐ZNF191‐NF (without C2H2 zinc finger domain) alone using Lipofectamine 2000 agents (Invitrogen). The luciferase activity of each transfection was examined with a dual reporter assay system (Promega) according to the manufacturer's protocol at 24 h after transfection. The plasmid pRL‐SV40 (Promega) encoding Renilla luciferase was applied as internal normalization for each transfection.

### 2.3 Chromatin immunoprecipitation (ChIP)‐PCR

Chromatin immunoprecipitation was performed in Hep3B cells as described previously.^25^ Briefly, the crosslinked protein–DNA complexes were immunoprecipitated with a rabbit validated polyclonal anti‐ZNF191 antibody (ab176589, from Abcam) or rabbit IgG (Sigma‐Aldrich) as a negative control. Then isolated DNA was subjected to real‐time PCR. The primers are listed in Table S1.

### 2.4 Electrophoretic mobility shift assay (EMSA)

ZNF191 recombinant proteins were purified as described previously,^26^ and 5’ biotin‐labeled oligonucleotides were synthesized and labeled in Invitrogen, China. DNA binding activity of the synthesized oligonucleotides to ZNF191 proteins was tested with a LightShift Chemiluminescent EMSA kit (Pierce). Unlabeled oligonucleotides were included in the binding reaction for competition assays. The sequences of the oligonucleotides are listed in Table S1.

### 2.5 Methylation array

Illumina Human Methylation 850K microarray profiling and data analysis were performed by Oebiotech. Briefly, genomic DNA was extracted from Hep3B and PLC/PRF/5 cells transfecting with lentivirus ShZNF191 (Sh‐ZNF191 group, including Sh‐ZNF191‐1 and Sh‐ZNF191‐2) or nonspecific control shRNAs (Sh‐NC group, including Sh‐NC1 and Sh‐NC2) and assayed with Methylation 850K BeadChip according to Illumina's protocol. Methylation levels were quantified with the *b* values, from 0 (unmethylated) to 1 (totally methylated). The value of average delta Beta (Δ*b* = Beta Sh‐ZNF191 – Beta Sh‐NC) < 0.1 and the associated *p* < 0.05 were considered significant for screening differential methylation sites (DMS). Then the pathways in which DMS significantly enriched were identified with Gene Ontology (GO) term enrichment analysis and Kyoto Encyclopedia of Genes and Genomes (KEGG) pathway analysis.

Additional experimental methods are described in Document S1.

## 3 RESULTS

### 3.1 DNMT1 is upregulated in human HCCs and correlated with poorer survival

**FIGURE 1:**
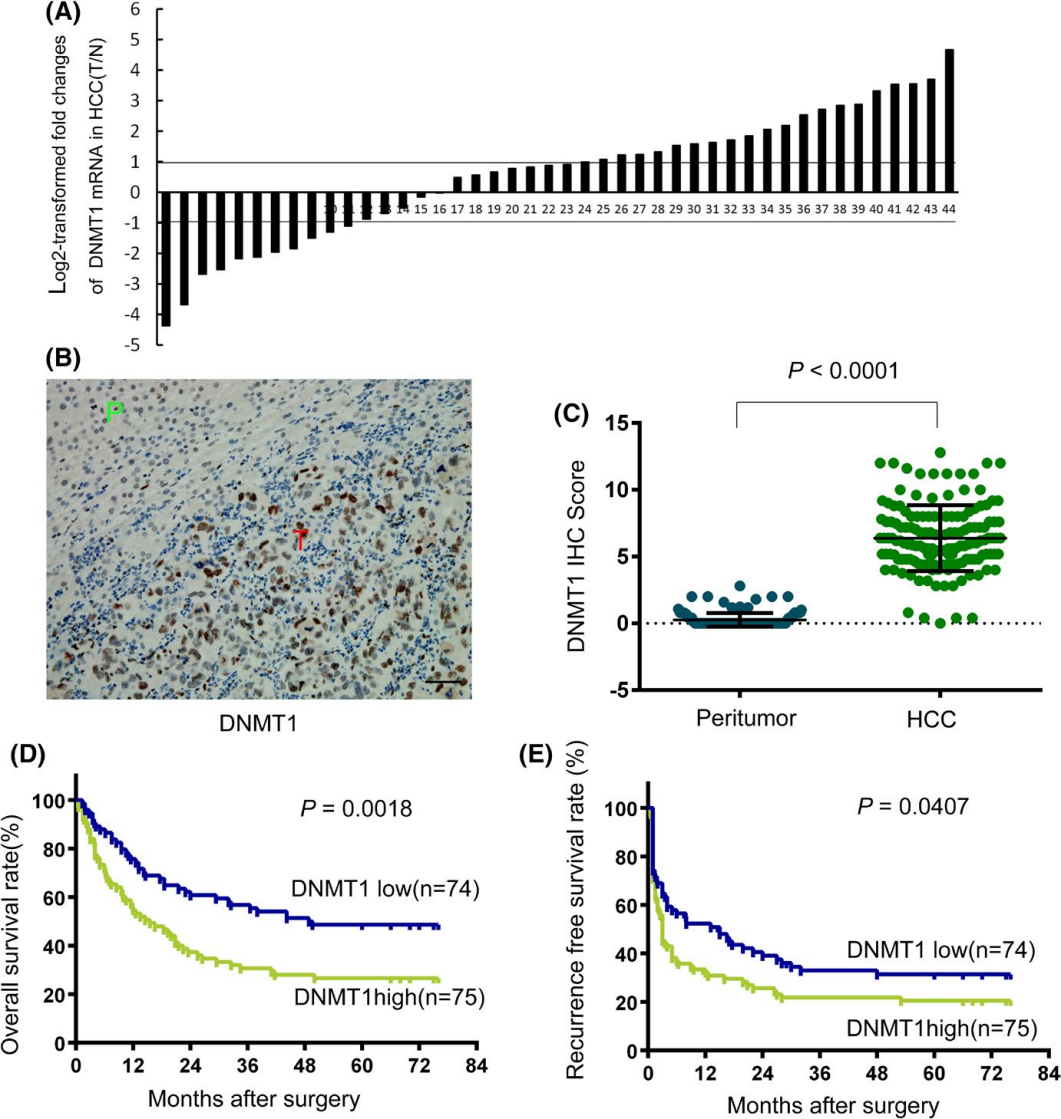
Upregulation of DNMT1 in HCCs is correlated with poor patient prognosis. (A) mRNA expression levels of DNMT1 were measured in 44 paired HCCs (nos. 1–44, numbered from relative low to high expression level) using real‐time PCR and the 2^−ΔΔCt^ method. (B) Representative DNMT1 immunohistochemistry (IHC) staining in tumor tissue (T) and adjacent peritumor tissue (P) of HCC specimens. Scale bar = 50 μm. (C) Scatter plots for corresponding evaluated DNMT1 IHC score in tumor and peritumor tissues (*n* = 149). IHC scores were compared by the Student's *t*‐test. (D, E) The Kaplan–Meier plots indicate the overall survival rate (D) and recurrence‐free survival rate (E) for HCC patients categorized by DNMT1 expression. *p* value is determined by the log‐rank test

To investigate the expression status of DNMT1 in HCCs, DNMT1 mRNA expression between 44 pairs of fresh HCC tissues (T) with their adjacent nontumor tissues (N) was compared by real‐time PCR assay and the 2^−ΔΔCt^ method. As shown in Figure 1A, 22 of 44 (50.0%) cases showed significant upregulation of DNMT1 in HCC, 22 of 44 (50.0%) cases showed no alteration or reduction. Moreover, the upregulation of DNMT1 mRNA in HCC tissues was further supported in three different datasets of the Oncomine database (Figure S1A).

Next, we examined DNMT1 protein expression in tissue microarrays (TMAs), which contained 149 pairs of human HCC specimens and adjacent nontumor tissues by immunohistochemistry (IHC) staining (Figure S2). As shown in Figure 1B, DNMT1 was highly expressed in the nuclei of tumor cells, while DNMT1 was lowly expressed in adjacent liver cells. In agreement with the higher mRNA level of DNMT1 in HCC, the frequency of positive staining DNMT1 was significantly higher in tumors (75/149; 50.3%) than that in nontumor tissues (74/149; 49.7%; *p* < 0.0001) (Figure 1C).

The clinicopathological analysis demonstrated that high expression of DNMT1 protein was closely correlated with younger age (*p* = 0.017), high AFP level (*p* = 0.011), tumor venous invasion (*p* < 0.001), and a high BCLC (the Barcelona Clinic Liver Cancer staging) stage (*p* = 0.001) with the *χ* ^2^ test (Table S2). Despite the Cox proportional hazards model showed that high DNMT1 expression was not an independent prognostic factor with respect to overall survival (OR) and recurrence‐free survival (RFS) (Tables S3 and S4), the Kaplan–Meier survival analysis revealed that patients with high DNMT1 expression had significantly worse OR (mean of 28.76 vs. 42.61 months, log‐rank test *p* = 0.0018; Figure 1D) and RFS (mean of 19.34 vs. 25.35 months, log‐rank test *p* = 0.0407; Figure 1E) than those with low DNMT1 expression. Furthermore, patients with higher expression of DNMT1 mRNA had significantly worse OS in two public databases including DriverDBv3 (totally 158 cases) and the Kaplan–Meier Plotter (totally 365 cases) as well (Figure S1B). Taken together, the data suggest that DNMT1 overexpression is frequently detected in HCCs and that high DNMT1 expression is associated with poor patient prognosis.

### 3.2 ZNF191 positively regulates DNMT1 expression in hepatoma cells

Next to search for the mechanism of the upregulation of DNMT1, we first studied the status of mutation or copy number alteration of *DNMT1* gene in HCCs in the Cancer Genome Atlas (TCGA) database. However, only 15 mutations (0.8%) are presented in 1608 HCC patients/1680 samples of 9 cohorts (Figure S3A,B) in the database. Thus we considered that the upregulation of DNMT1 mRNA in HCCs does not result from *DNMT1* gene mutation or copy number alteration.

Combining the data of ChIP‐sequencing that ZNF191 protein can bind to *DNMT1* gene promoter (2000 bp upstream of transcription start site) both in HEK‐293T and Hep3B cells (please see supporting Tables S6 and S7 of our previous study^24^), and the transcription data that DNMT1 mRNA expression level is downregulated (0.5‐fold) with ZNF191 transient knockdown in human hepatic cell L02,^23^ we supposed the *DNMT1* gene is a potential target gene of ZNF191.

**FIGURE 2:**
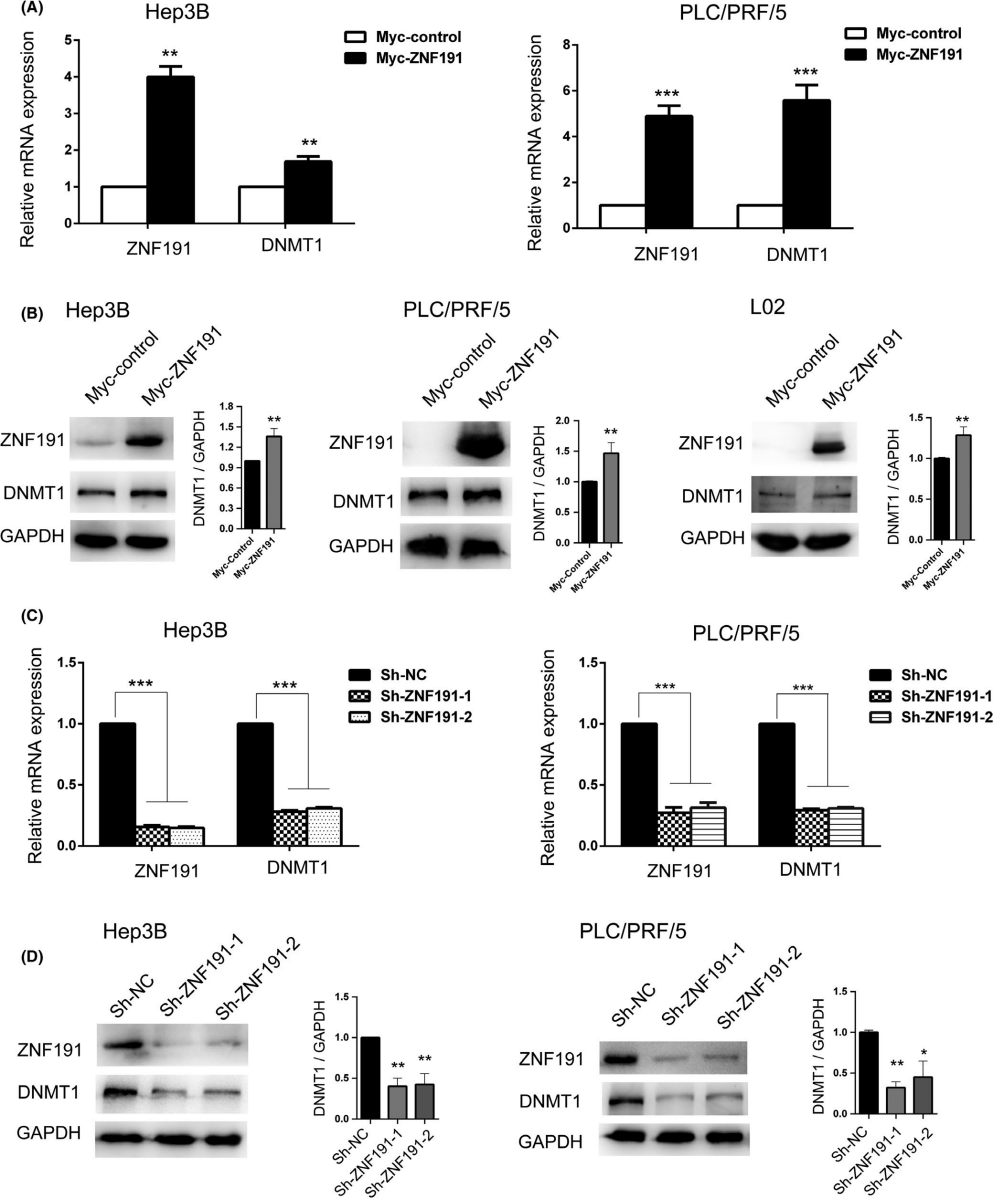
ZNF191 can positively regulate DNMT1 expression in hepatoma cells. (A) Ectopic overexpression of ZNF191 induced DNMT1 mRNA expression in hepatoma cell line Hep3B (left panel) and PLC/PRF/5 (right panel) measured with qRT‐PCR. (B) DNMT1 protein expression was analyzed with western blotting in Hep3B, PLC/PRF/5, and human liver cell L02 with transient ZNF191 overexpression. Quantitative densitometric analysis of DNMT1 normalized to GAPDH using ImageJ software (right panel). (C, D) Stable knockdown of ZNF191 downregulated DNMT1 mRNA (C) and protein expression (D) with indicated lentivirus in Hep3B and PLC/PRF/5. *p < 0.05, **p < 0.01, ***p < 0.001

To study the relationship of ZNF191 and DNMT1 in hepatoma cells, we analyzed the DNMT1 mRNA and protein expression in hepatoma cell Hep3B and PLC/PRF/5 with transient overexpressed ZNF191. As expected, DNMT mRNA and protein levels were upregulated with ectopic ZNF191 overexpression (Figure 2A,B). Consistently, DNMT1 protein expression is also positively induced in L02 cell with ZNF191 overexpression (Figure 2B, right panel). Moreover, in stable ZNF191 knockdown Hep3B cells and PLC/PRF/5 with shRNA, the expression of DNMT1 mRNA and protein decreased as the endogenous ZNF191 protein depleted when compared with controls (Figure 2C,D).

### 3.3 ZNF191 activates the *DNMT1* promoter and directly binds to the promoter

**FIGURE 3:**
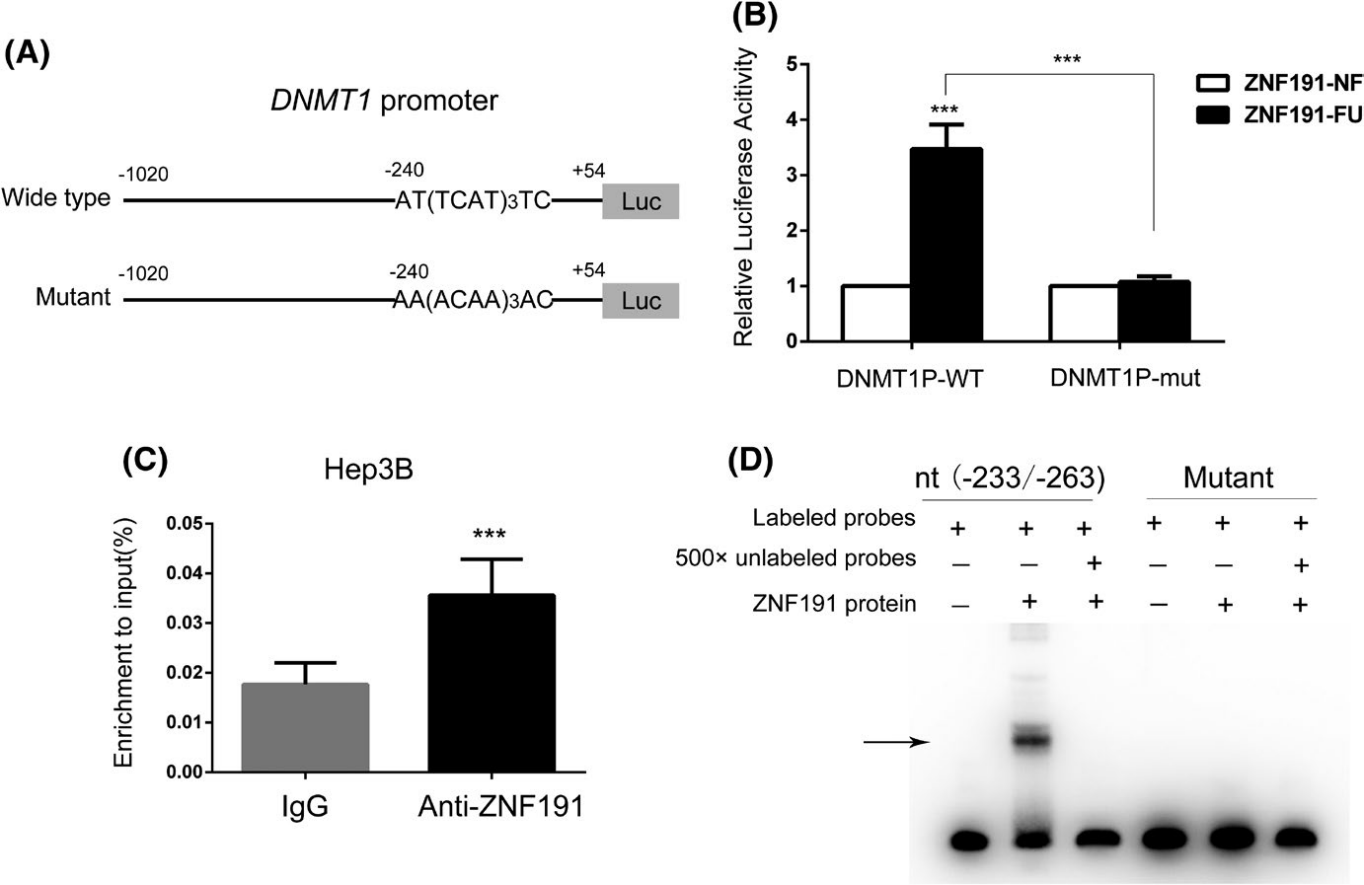
ZNF191 activates *DNMT1* transcription and directly binds to *DNMT1* promoter. (A) The sketch of 5’ flanking region (−1020/+54) of the *DNMT1* gene, and potential ZNF191 binding site and the mutated sequence for further analysis. (B) The transcriptional activation effect of ZNF191 on *DNMT1* promoters. Activity of wild‐type *DNMT1* promoter and the mutant was tested with luciferase reporter assays. ZNF191‐NF (without C2H2 zinc finger domain) served as negative controls. (C) The binding ability of ZNF191 protein to the *DNMT1* promoter was measured with ChIP‐qPCR. The primers specific to the region nucleotide (nt) −205/−342 containing the potential ZNF191 binding sequence AT(TCAT)_3_TC. ***p < 0.001. (D) EMSA was used to detect DNA binding activity of purified ZNF191 protein to 30‐bp annealed oligonucleotides of *DNMT1* promoter and its mutant. Arrow indicates the specific band of ZNF191 protein binding to the oligonucleotides

Since ZNF191 can positively regulate both mRNA and protein levels of DNMT1, and ZNF191 protein can bind to *DNMT1* gene,^24^ next we analyzed whether ZNF191 can directly bind to the *DNMT1* promoter and increase its transcription activity. With close analysis of the 5’ flanking region (−3000/+100) sequences of the *DNMT1* gene, we discovered a potential ZNF191 binding site at nt‐240 with the sequences AT(TCAT)_3_TC (Figure 3A, Figure S3C), which are the typical TCAT repeats, the key binding sequences of ZNF191 in vivo as we previously reported.^24^ Promoter luciferase assay showed that full length of ZNF191 (ZNF191‐FU) can increase the transcription activity of the 1 Kbps *DNMT1* promoter by about 3.5‐fold compared with transfecting negative control truncated ZNF191 (ZNF191‐NF, without C2H2 zinc finger domain) vector. When the AT(TCAT)_3_TC sequences were mutated to AA(ACAA)_3_AC (Figure 3A, bottom panel), the activation ability of mutated *DNMT1* promoter by ZNF191 was remarkably decreased compared with that of wild‐type promoter (Figure 3A,B).

Furthermore, ChIP‐qPCR verified that endogenous ZNF191 protein was enriched around *DNMT1* promoter in Hep3B cells (Figure 3C). EMSA further confirmed that recombinant ZNF191 protein could directly bind to the sequences AT(TCAT)_3_TC with high affinity (Figure 3D). While the binding sequences were mutated, ZNF191 had no binding capacity accordingly (Figure 3D). Thus, the data suggest that ZNF191 protein can bind to the *DNMT1* promoter directly, and the key binding sequences are at nt‐240 AT(TCAT)_3_TC.

### 3.4 DNMT1 and ZNF191 expression correlate positively in HCCs

**FIGURE 4:**
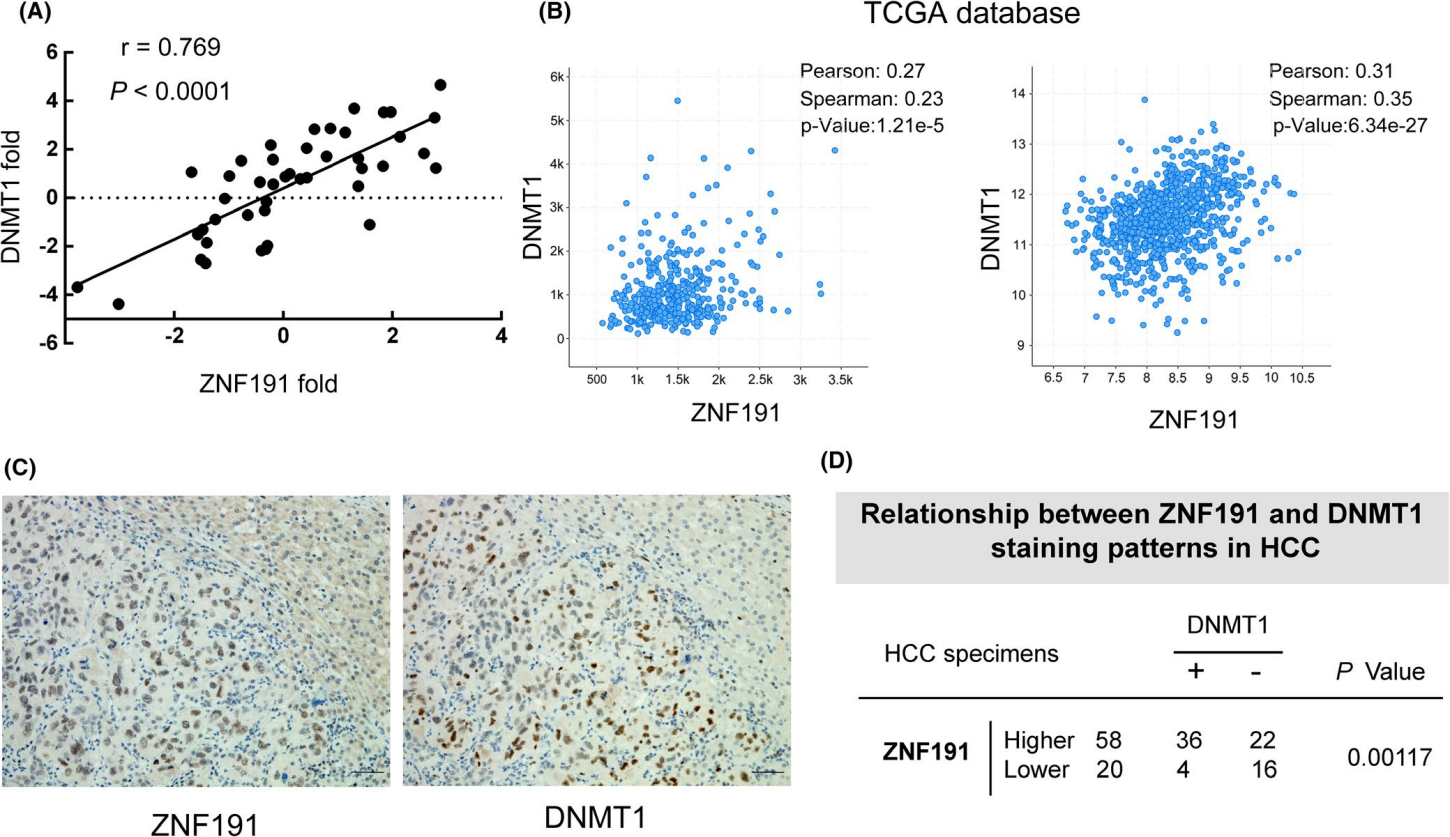
Correlation between the expression of ZNF191 and DNMT1 in human HCCs. (A) The mRNA level of ZNF191 and DNMT1 in 44 pairs of HCC tissues and paracancerous tissues was measured with qRT‐PCR. Pearson's correlation test was performed to analyze the correlation. (B) ZNF191 and DNMT1 mRNA expression levels were correlated in 373 HCC specimens (left panel) and 967 cancer cell lines (right panel). Data were obtained from The Cancer Genome Atlas (TCGA) database at the cbioportal website (http://www.cbioportal.org). Unit of axis: RNA‐sequencing V2 RSEM. Pearson's and Spearman's correlation coefficients are listed in the panels. (C) Representative ZNF191 and DNMT1 IHC staining in consecutive sections of HCC specimen. Scale bar = 50 μm. D, The relationship between ZNF191 and DNMT1 staining status in 78 HCC specimens. *p* value was determined by the *χ* ^2^ test (right panel)

To further verify the correlation of ZNF191 and DNMT1 in HCCs in vivo, we first studied the correlations of the ZNF191 mRNA expression level with DNMT1 in the 44 human HCC tissues. Fold changes of ZNF191 and DNMT1 mRNA expression in HCCs compared to adjacent nontumor tissues were measured with qRT‐PCR. Then correlation analysis with Pearson's correlation test confirmed that DNMT1 expression was significantly correlated with ZNF191 expression (*r* = 0.769; *p* < 0.001, Figure 4A). Moreover, their mRNA levels correlation was further confirmed in a cohort of 373 HCC samples (*r* = 0.27; *p* < 0.001) and 967 cancer cell lines (*r* = 0.31; *p* < 0.001) of TCGA dataset (Figure 4B).

Next the correlation between the ZNF191 and DNMT1 proteins expression in consecutive sections of 78 HCC specimens was examined with IHC staining (Figure 4C). As shown in Figure 4D, DNMT1 protein expression was positively correlated with ZNF191 expression (*χ* ^2^ test, *p* = 0.00117). Taken together, the data demonstrated that DNMT1 expression was correlated with that of ZNF191, and they were clinically relevant in human HCCs.

### 3.5 ZNF191 alters DNA methylation in hepatoma cells and DMS enrich in the PI3K‐AKT pathway

**FIGURE 5:**
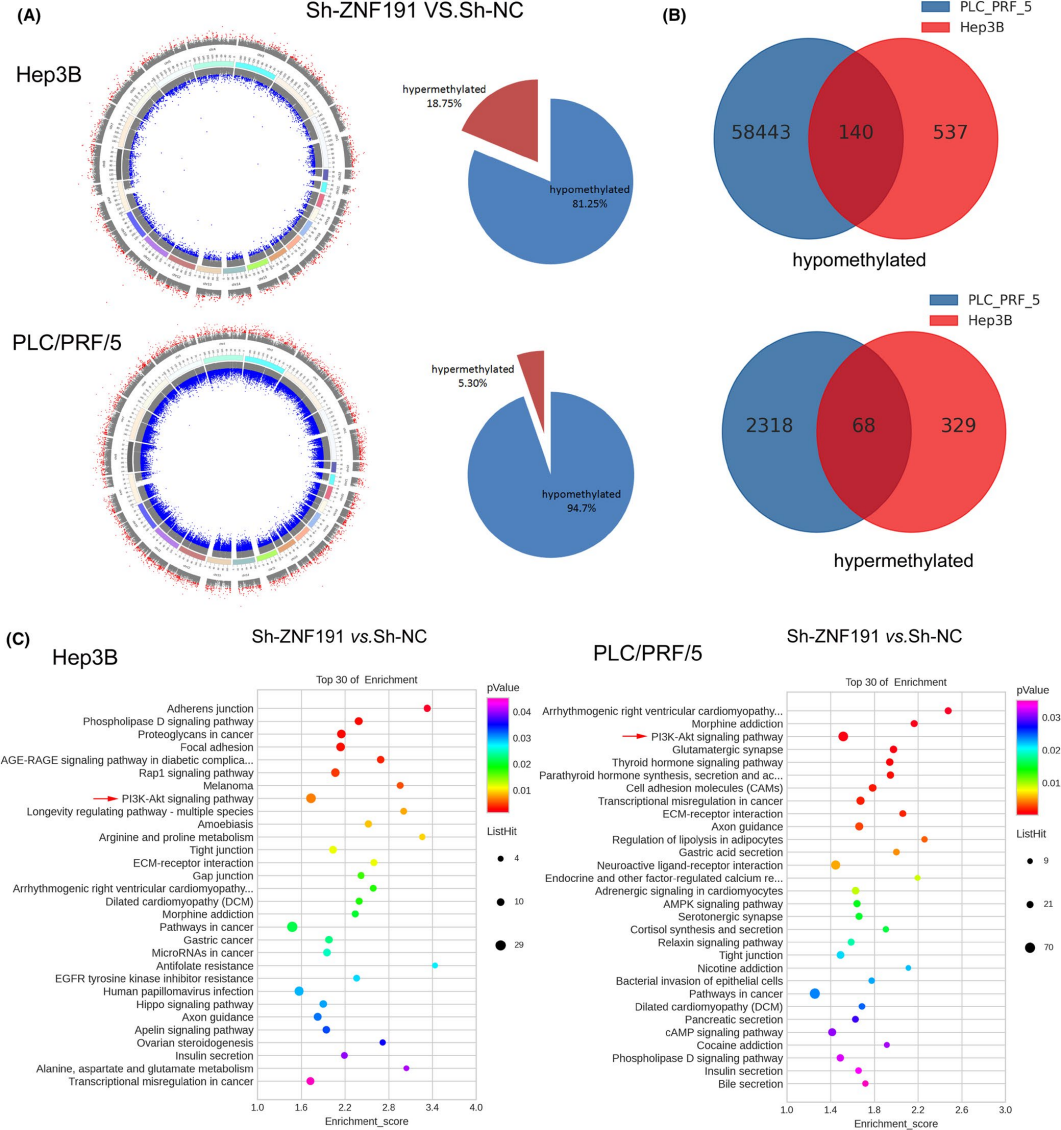
DNA methylation alteration in Hep3B and PLC/PRF/5 cells with ZNF191 knockdown. (A) The distribution of delta Beta (∆*b*) values of DMS on the human genome UCSC hg19 (left panel) and the proportion of hypermethylated and hypomethylated DMS (right panel) in ZNF191 stable knockdown Hep3B and PLC/PRF/5 cells compared with controls. The outer ring indicates that ∆*b* > 0, and the inner ring indicates that ∆*b* < 0, where gray indicates non‐DMS, red indicates high methylation sites, and blue indicates low methylation sites. (B) Venn diagram of hypermethylated and hypomethylated DMS in Hep3B and PLC/PRF/5 cells with ZNF191 stable knockdown. (C) KEGG pathway enrichment analysis of the DMS. Red arrows indicate DMS are enriched in the PI3K‐AKT pathway both in Hep3B and PLC/PRF/5 cells (Sh‐ZNF191 vs. Sh‐NC)

Since DNMT1 is the foremost contributor in the mammalian DNA methylation machinery,^15^ and ZNF191 can transcriptionally regulate *DNMT1* directly, we wondered whether ZNF191 can alter DNA methylation in hepatoma cells. With Illumina Human Methylation 850K microarray, we found Hep3B and PLC/PRF/5 cells with stable ZNF191 knockdown had remarkable alteration of DNA methylation with the hypomethylated DMS accounting for the most proportion (81.25% and 94.70%, respectively, Figure 5A) compared with those of hypermethylated DMS (18.75% and 5.3%, respectively, Figure 5A), despite that DMS in common are not significant in both cells (Figure 5B).

Furthermore, GO enrichment analysis demonstrated DMS in ZNF191 knockdown hepatoma cells are involved in biological processes of regulating cell–cell signaling, cell adhesion, intracellular signal transduction, etc. (Figure S4). Most interesting, using KEGG pathway analysis, we identified DMS are enriched in the PI3K‐AKT pathway both in Hep3B and PLC/PRF/5 cells with ZNF191 knockdown (Figure 5C and Table S5).

Thus, ZNF191 can alter DNA methylation of hepatoma cell, and DMS are involved in different physiological and pathophysiological processes and different pathways especially the PI3K‐AKT pathway.

### 3.6 DNMT1 contributes the effect of ZNF191 on hepatoma cell proliferation via the PI3K‐AKT pathway

**FIGURE 6:**
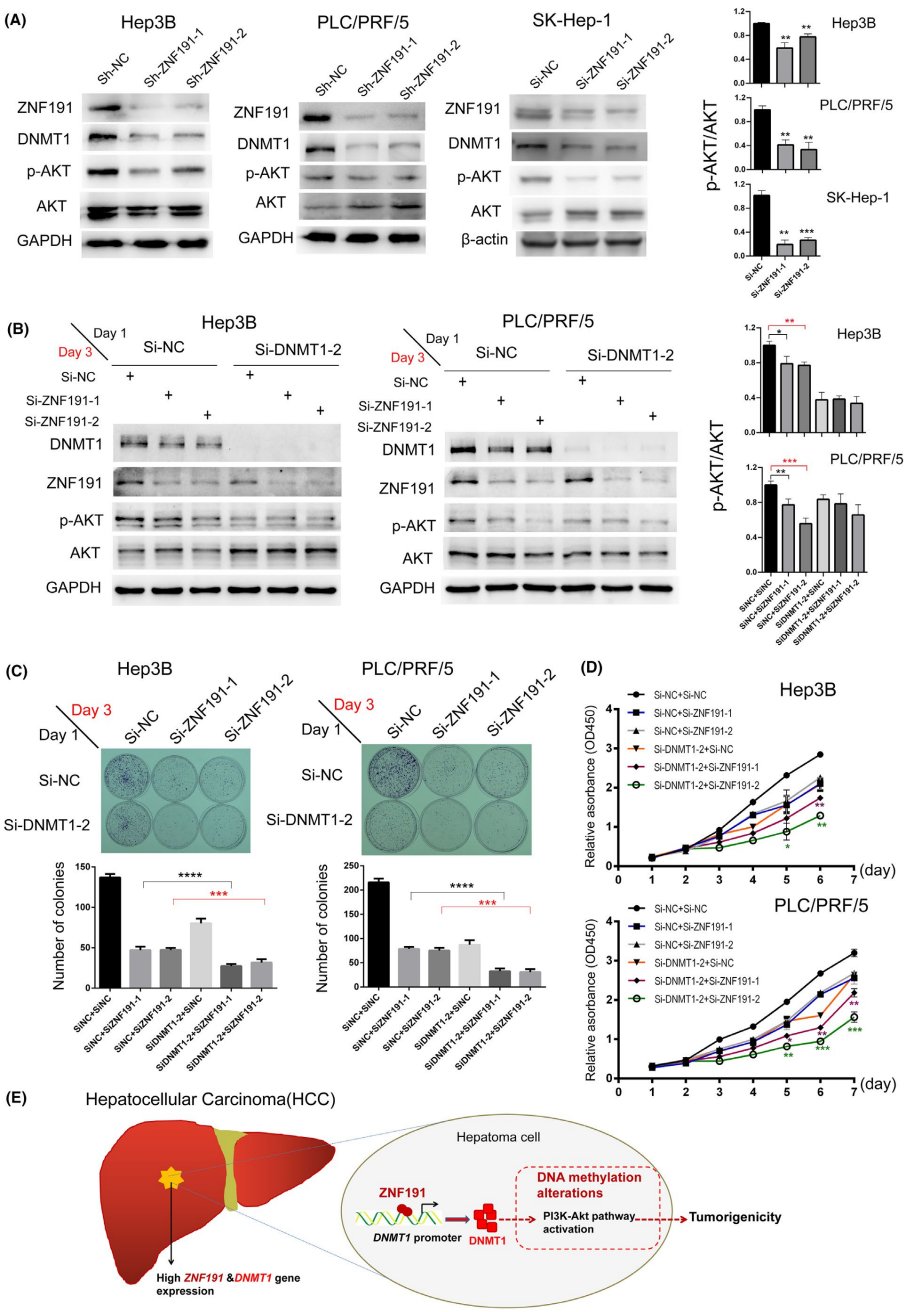
DNMT1 mediates the effect of ZNF191 on hepatoma cell proliferation through AKT pathway. (A) Western blotting analysis of DNMT1, p‐AKT (Ser473), and AKT protein expression in Hep3B and PLC/PRF/5 cells with stable ZNF191 knockdown, as well as in SK‐Hep‐1 with transient ZNF191 knockdown. Quantitative densitometric analysis of p‐AKT compared with total AKT protein using ImageJ software (right panel). (B) Western blotting analysis of the above proteins in Hep3B and PLC/PRF/5 with double knockdown of DNMT1 and ZNF191. Quantitative analysis of p‐AKT compared to total AKT protein expression (right panel). (C, D) Effect of double knockdown of DNMT1 and ZNF191 on cell proliferation of Hep3B and PLC/PRF/5 was measured with the colony formation assay (C), and CCK‐8 assay (D). (E) The hypothetical working model. The diagram indicates ZNF191 can transactivate *DNMT1*, alter DNA methylation, and subsequently activate the PI3K‐AKT pathway to promote hepatoma cells proliferation. *p < 0.05, **p < 0.01, ***p < 0.001

The finding that ZNF191 can alter the DNA methylation of genes involved the PI3K‐AKT pathway prompted us to determine how ZNF191 affects the signaling pathway in hepatoma cells. With western blotting, we found that p‐AKT (phosphorylated Akt) protein, the key activated protein of PI3K‐AKT pathway,^27^ was downregulated significantly compared with total AKT protein when the endogenous ZNF191 was depleted in Hep3B and PLC/PRF/5 cells (Figure 6A). Similar reduction in p‐AKT expression along with DNMT1 was found in hepatoma cell line SK‐Hep‐1 with transient ZNF191 knockdown (Figure 6A).

Next we tested whether this ZNF191 activation of AKT pathway in hepatoma cells is DNMT1‐dependent. When endogenous DNMT1 was depleted with siRNA in Hep3B and PLC/PRF/5, we found ZNF191 knockdown no longer significantly reduced the p‐AKT protein level compared with ZNF191 knockdown alone without DNMT1 depletion beforehand (Figure 6B). Thus ZNF191 activation of AKT pathway is DNMT1‐ dependent.

Given that the PI3K‐AKT pathway could promote cell proliferation,^28^ and ZNF191 promotes HCC cell growth in early HCC as we previously reported,^23^ we wondered whether the cell proliferative function of ZNF191 is partially DNMT1‐dependent via activating AKT pathway. Therefore, we examined the effect of ZNF191 on hepatoma cell growth with or without endogenous DNMT1 protein. As shown in Figure 6C,D, depleting ZNF191 induced a remarkable reduction in number of colonies in DNMT1 knockdown cells. Consistently, Cell Counting Kit‐8 (CCK‐8) assay showed that double depletion of DNMT1 and ZNF191 inhibited cell proliferation more obviously as well. Thus we identified that ZNF191/DNMT1/p‐AKT axis can promote hepatoma proliferation.

Taken together, ZNF191 may function through upregulating DNMT1, regulating DNA methylation of genes involved in the PI3‐AKT pathway and activating the signaling to promote hepatoma cell proliferation (Figure 6E).

## 4 DISCUSSION

DNMT1, a well‐documented oncoprotein, is essential for maintaining DNA methylation patterns during carcinogenesis, and plays important roles in tumor cell growth, and serves as a potential therapeutic target in several human cancers including HCC.^29^, ^30^, ^31^ In adult human liver tissue, DNMT1 mRNA is lowly expressed (https://www.ncbi.nlm.nih.gov/gene/1786). However, DNMT1 mRNA and protein expression are significantly higher in HCCs, and are associated with worse patient outcome (Figure 1). Our findings are consistent with the previous studies.^6^, ^10^, ^11^, ^12^, ^13^, ^14^

Since *DNMT1* gene mutations or copy number alterations are rare in HCCs (Figure S3A,B), we focused on the transcriptional regulation of *DNMT1*. In this study, we identified that transcription factor ZNF191 can positively regulate DNMT1 expression in hepatoma cells (Figure 2). With a series of assays (Figure 3), ZNF191 is confirmed to bind to *DNMT1* promoter directly and transactivate *DNMT1* gene, and the key binding sequence was at *DNMT1* nt‐240 (ATTCATTCATTCATTC), which is not overlapped with binding sequences of previously reported transcriptional factors P53, SP1, E2F, AP1, and ERE1/2,^16^, ^18^, ^19^, ^20^ or target sequences of miRNA regulators such as miR‐185, miR‐148a‐3p, and microRNA‐140.^32^, ^33^, ^34^ Moreover, DNMT1 and ZNF191 expression were correlated in HCCs (Figure 4). Thus, we identified a novel mode of transcriptional regulation mechanism of DNMT1 abundance in HCCs.

Next, through methylation array analysis, we found ZNF191 stable knockdown can change DNA methylation in hepatoma cells. Since DNA methylation is regulated via three DNMTs (DNMT1, DNMT3A, and DNMT3B) cooperatively,^15^, ^35^, ^36^ it is possible DNMT3A and DNMT3B (the de novo methyltransferases), and their cofactor DNMT3L^35^ are involved in the DNA methylation alteration. However, mRNA expression of DNMT3A, DNMT3B, and DNMT3L was not correlated with ZNF191 expression in 967 cancer cell lines and HCC tissues (373 samples) of TCGA database compared with that of DNMT1 with ZNF191 expression (Tables S6 and S7), and ZNF191 protein is not enriched in the promoter of the three genes (Tables S6 and S7 of our previous study^24^). Thus combining the data of TCGA, ChIP‐Seq,^24^ and expression profile data,^23^ we consider the ZNF191 induction of DNA methylation alteration is mainly via DNMT1.

Previous studies report DNMT1 can hypermethylate the promoters of TSG genes such as *PTEN* and *MTs* (metallothionein genes) to promote HCC cell growth via DNMT1/PTEN/Akt pathway^32^ and DNMT1/MT/NF‐κB pathway,^34^ respectively. In this study, we found several DMS of *PTEN* and *MTs* in either Hep3B or PLC/PRF/5 cells with ZNF191 knockdown with methylation array (Table S8). However, ZNF191 stable knockdown did not alter PTEN and MT1 protein expression significantly, despite that ZNF191 regulates DNMT1 expression. The reason for the latter phenomenon remains unknown. It is possible that ZNF191 might influence PTEN and MT1 through other mechanisms. Nonetheless, we identified DMS are enriched in the PI3K‐AKT pathway in both cell lines (Table S5 and Figure 5), and proved ZNF191/DNMT1/p‐AKT axis promote hepatoma cell growth (Figure 6). We suppose the activation of PI3K‐AKT signaling by ZNF191 may be the combined effects of the multiple gene methylation alteration (Table S5), which warrants further investigation.

Furthermore, we also found DMS in a number of hypomethylated tumor‐promoting gene (including *uPA*, *HPA*, *TFF3*, *MAT2A*, etc.) and frequently methylated TSGs (*DLC1*, *CHD5*, *IGFBP2*, *ADAM23*, *LRRC4*, *LASS2*, *SCAI*, *HOXB13*, *CSRNP1*, *RASSF1A*, *RB1*, *GSTP1*, *NQO1*, *PROX1*, *NORE1B*, *RIZ1*, *RELN*, etc.) which identified in primary human HCC,^4^, ^11^ either in Hep3B cell or PLC/PRF/5 with ZNF191 knockdown. Of note, like DMS of *PTEN* and *MTs*, few of them exist in both cell lines when taken the intersection. We suppose the reasons for the paradox may be from two aspects. First, different cell lines with ZNF191 knockdown have different DMS; second, the methylation status of hepatoma cells is different from HCC tissues, since methylation in HCC occurs in a gene‐specific and disease‐specific manner as Nishida et al demonstrated.^8^ Certainly, the precise mechanism of the phenomenon needs further study.

ZNF191 plays complicated role in HCCs via activating different target genes.^24^ We have demonstrated that ZNF191 transactivates *WNT8B* and *CTNNB1* in HCC to promote cell proliferation.^23^, ^25^ In this study, we found that ZNF191 directly transactivates *DNMT1*. Thus the pro‐proliferation effect of ZNF191 may be driven by combination of two different mechanisms including canonical Wnt pathway and PI3K‐AKT pathway (Figure S5), in addition to its anti‐metastasis effect at late stage of HCCs.^24^

The present study has some limitations. First, we have identified dozens of DMS enriched in the PI3K‐AKT pathway in hepatoma cells with ZNF191 knockdown (Table S5) in this study. However, certain essential DNMT1 targets, which are responsible for the mechanistic link between ZNF191 and AKT phosphorylation, remain to be determined and need further investigation. Second, we only analyzed ZNF191/DNMT1/p‐AKT axis in hepatoma cells (Figure 6E), thus our proposed signal pathway regulation requires further validation in mouse models of liver cancer and HCC patients.

Taken together, our findings suggest the pro‐proliferation effect of ZNF191/DNMT1/p‐AKT axis in hepatoma cells, which implies ZNF191 status in HCC tissues may affect the therapeutic effect of PI3K inhibitors^3^ and DNMTs inhibitors,^31^ and warrants further study to guarantee precise treatment of the disease.

## CONFLICT OF INTEREST

The authors have no conflict of interest.

## AUTHOR CONTRIBUTIONS

Guoyuan Liu, Xiuping Liu, Wei Jiang, and Cheng Huang conceptualized and supervised the study. Yufeng Liu, Hanghang Cheng, Chenchen Cheng, Fengyun Zheng, Zhonghua Zhao, Qi Chen, and Wenjiao Zeng performed experiments. Yufeng Liu and Hanghang Cheng performed bioinformatic analysis. Cheng Huang provided the clinic samples. Guoyuan Liu and Xiuping Liu wrote the manuscript.

## Supporting information

Fig S1‐S5

Table S1‐S8

Supplementary Material

## Data Availability

The datasets used and analyzed during the current study are available from the corresponding author upon reasonable request.
