# Supplementary material for: ZNF191 alters DNA methylation and activates the PI3K‐AKT pathway in hepatoma cells via transcriptional regulation of DNMT1
Source: Cancer Med. 2022 Jan 28;11(5):1269–80. doi: 10.1002/cam4.4535 (PMC8894703; doi:10.1002/cam4.4535)
Supplement: Supplementary file 1 — Fig S1‐S5 [file CAM4-11-1269-s001.pdf]

Supplemental figures

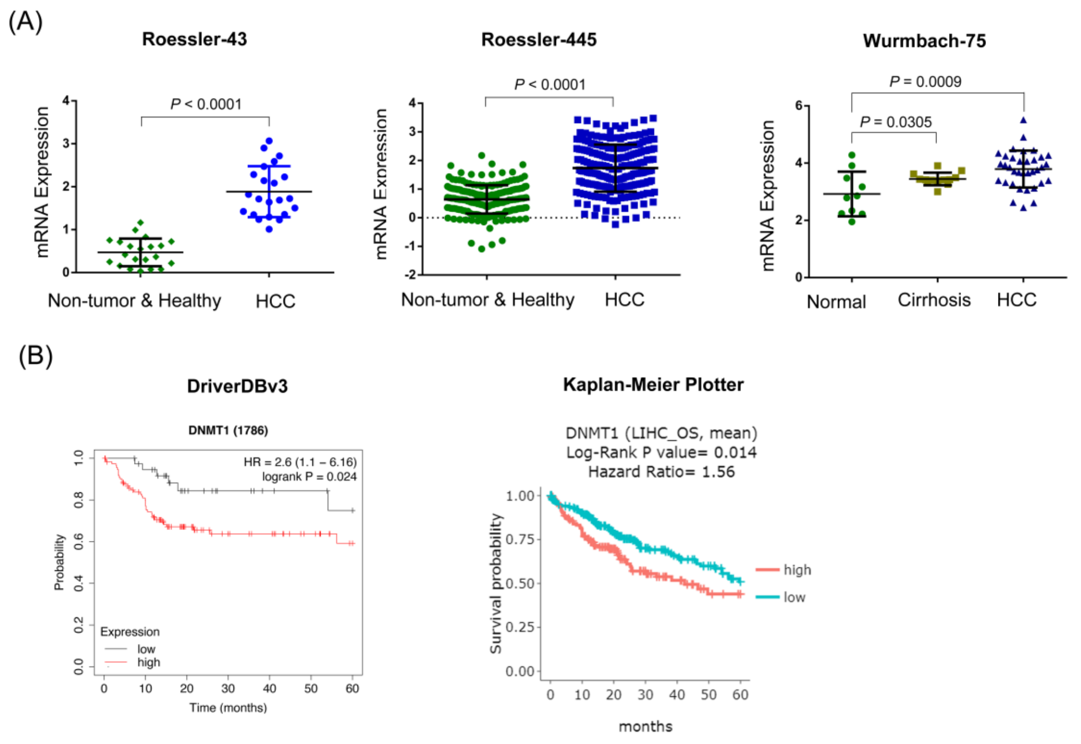

Figure S1. DNMT1 mRNA was upregulated in HCC tissues and correlated with poorer prognosis of the patients in public database. A, DNMT1 mRNA was upregulated in HCC tissues compared with nontumor/healthy liver tissues or cirrhosis tissues in 3 different datasets (Roessler-43, Roessler-445 and Wurmbach-75) from the Oncomine database (http://www.oncomine.org). B, Upregulation of DNMT1 mRNA in HCCs is correlated with poor patient survival in 2 public databases including DiverDBv3 (http://driverdb.tms.cmu.edu.tw) and Kaplan-Meier Plotter (https://kmplot.com/analysis/index.php?p=service).

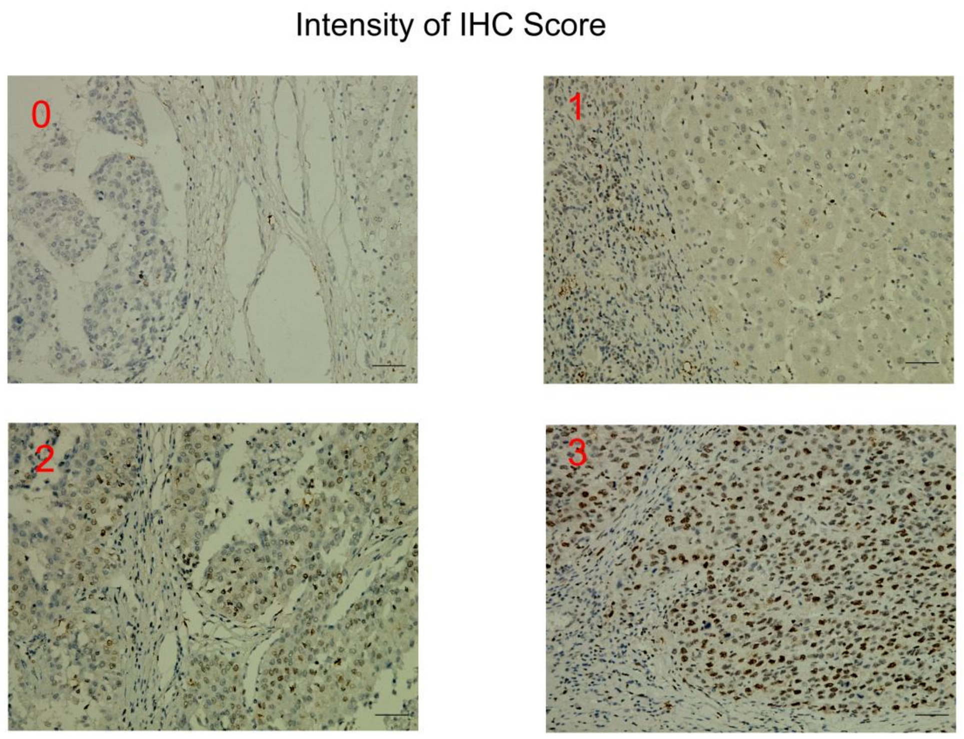

Figure S2. Representative immunohistochemistry (IHC) images with different intensity of DNMT1 staining. Intensity score, 0, negative; 1, weak; 2, medium; 3, strong. Scale bar = 100 μm.

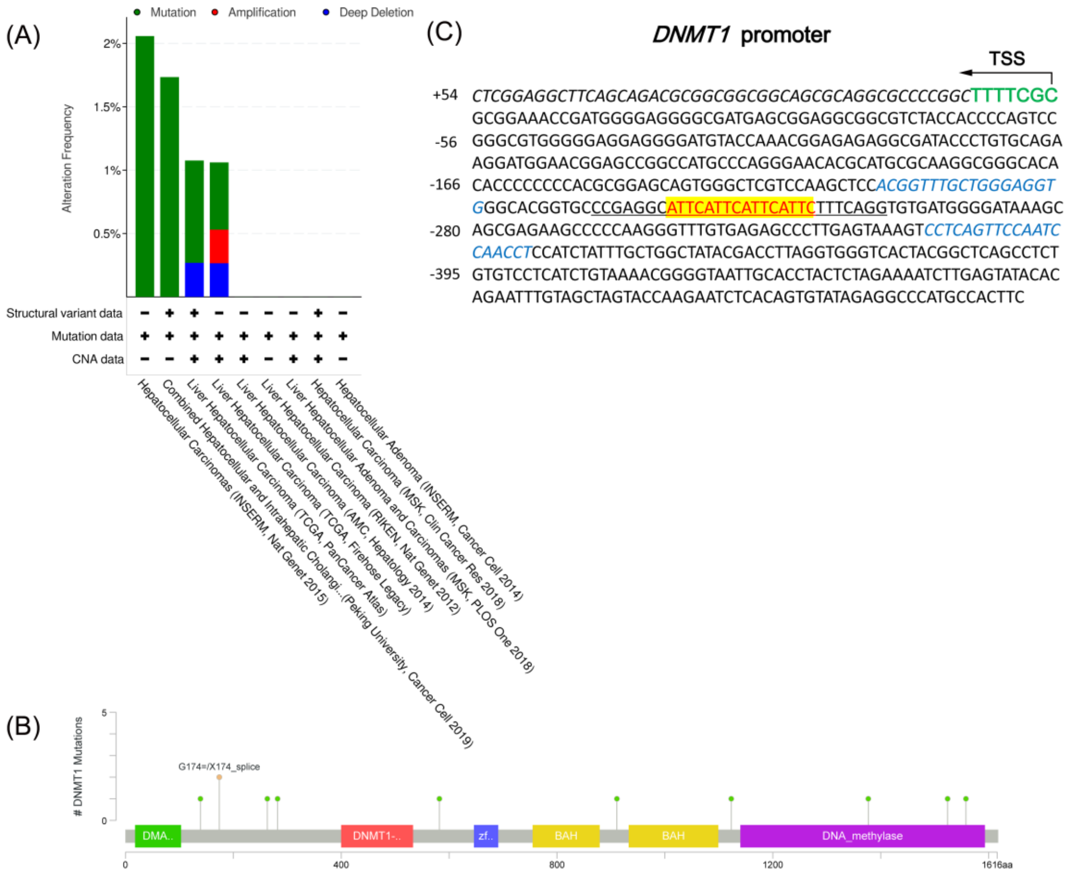

Figure S3. Mutations and copy number alteration of *DNMT1* in HCCs and the sequence of *DNMT1* promoter. A, Alteration frequency of *DNMT1* in 9 studies. B, 15 Mutations are found in 1608 patients / 1680 samples of 9 studies. The analysis was performed at the cbioportal website (http://www.cbioportal.org). Original data can be retrieved from TCGA database. C, The nucleotide sequences of the 5'-flanking region (-509/+54) of the *DNMT1* gene. The candidate ZNF191 binding sites are highlighted in yellow color. The sequences for primers used in ChIP-PCR are in blue color, and the sequences for primers used in EMSA are underlined. TSS, transcription start site.

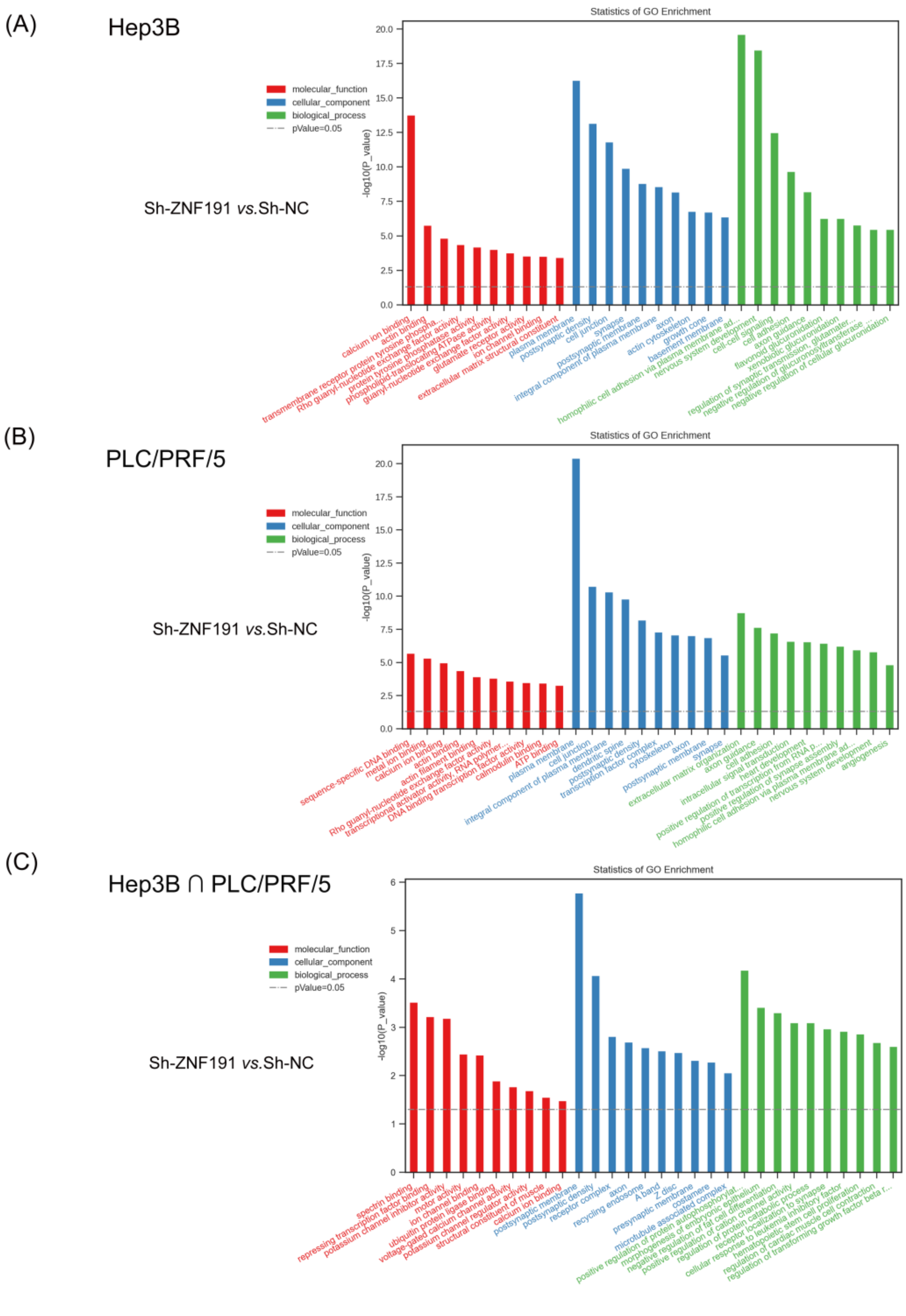

Figure S4. GO enrichment analysis of the DMS in Hep3B and PLC/PRF/5 cells (Sh-ZNF191 vs. Sh-NC).

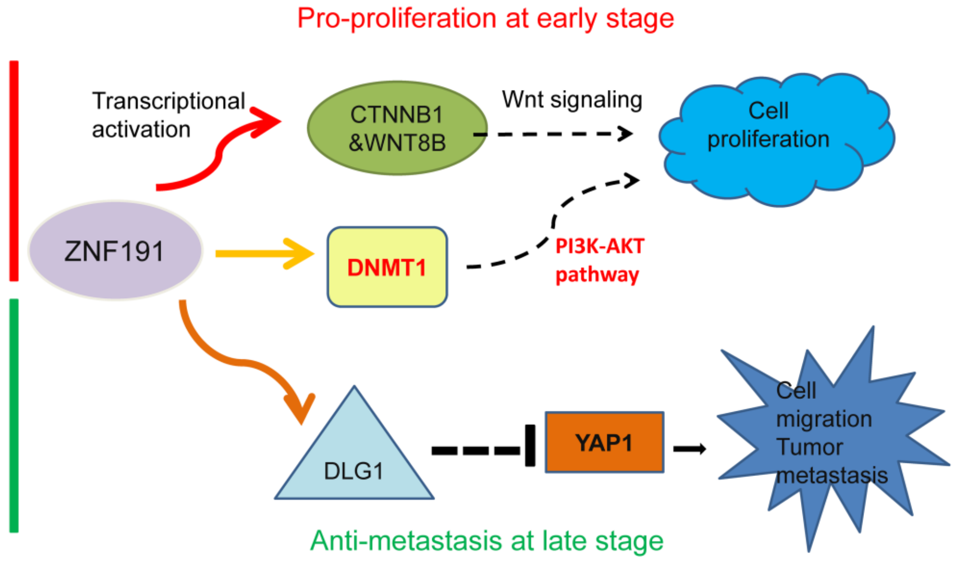

Figure S5. The working model of ZNF191 in hepatocarcinogenesis. This model diagram indicates complicated role of ZNF191 in different stage of HCC. Increased expression of ZNF191 at an early stage of HCC progression promotes cell proliferation via transactivating *DNMT1* and subsequently PI3K-AKT activation, in addition to canonical Wnt signaling activation.
