# Supplementary material for: ZNF191 alters DNA methylation and activates the PI3K‐AKT pathway in hepatoma cells via transcriptional regulation of DNMT1
Source: Cancer Med. 2022 Jan 28;11(5):1269–80. doi: 10.1002/cam4.4535 (PMC8894703; doi:10.1002/cam4.4535)
Supplement: Supplementary file 2 — Table S1‐S8 [file CAM4-11-1269-s002.pdf]

**Supplementary Table S1.** Sequence of small interference RNA, shRNA target sequences, and primers used for real-time RT-PCR, promoter construct, ChIP, and EMSA.

| | |
|---|---|
| **Small interference RNA** | |
| Si-NC | 5′-UUCUCCGAACGUGUCACGUTT-3′<br>5′- ACGUGACACGUUCGGAGAATT -3′ |
| Si-DNMT1-1 | 5′- GCACCUCAUUUGCCGAAUATT -3′<br>5′- UAUUCGGCAAAUGAGGUGCTT -3′ |
| Si-DNMT1-2 | 5′- GGGACUGUGUCUCUGUUAUTT -3′<br>5′- AUAACAGAGACACAGUCCCTT -3′ |
| Si-DNMT1-3 | 5′- GAGGCCUAUAAUGCAAAGATT -3′<br>5′- UCUUUGCAUUAUAGGCCUCTT -3″ |
| Si- ZNF191-1 | 5′- GCGAAGAGGGAUCAAGUAUTT -3′<br>5′- AUACUUGAUCCCUCUUCGCTT -3′ |
| Si-ZNF191-2 | 5′- GGAUUUGGAGAGUGAACUUTT -3′<br>5′- AAGUUCACUCUCCAAAUCCTT -3′ |
| Si-ZNF191-3 | 5′- GCAGUUUGUUGCCAUCCUATT -3′<br>5′- UAGGAUGGCAACAAACUGCTT -3 |
| **Target sequences of shRNA** | |
| Sh-NC | TTCTCCGAACGTGTCACGT |
| Sh-ZNF191-1 | GTGACAGTGCTGGAGGATT |
| Sh-ZNF191-3 | ATCGTATAGTCAAAGCTCA |
| **SYBR®Green real-time PCR primers** | |
| DNMT1 | F: 5’- CGGCTCTTCGGCAACATCC-3’<br>R: 5’- GCACTCTCTCGGGCTTTGG-3’ |
| ZNF191 | F: 5’-CTAATAGCATTGAGGTTG-3’<br>R: 5’-TCTCAGCCAAGGAGTATA-3’ |
| β2-MG | F: 5’-ATGAGTATGCCTGCCGTGTGAAC-3’<br>R: 5’-TGTGGAGCAACCTGCTCAGATAC-3’ |
| **Primers for DNMT1 promoter construct** | |
| DNMT1P(nt-1020/+54) | F:5’- atttctctatcgataggtaccCTCGGAGGCTTCAGCAGACG -3’<br>R:5'- gcttacttagatcgcagatctGAGATGGGGTATCCCTATGTTGC -3’ |
| Mut-DNMT1P | F:5’-CGAGGCAAACAAACAAACAAACTTTCAGGTGTGAT-3’<br>R:5'- GGCACCGTGCCCACCTCCCAGCAAACCGTGGA-3’ |
| **Probe sequence for EMSA** | |
| DNMT1(nt-240) | F: 5’- CCTGAAAGAATGAATGAATGAATGCCTCGG -3’<br>R: 5’- CCGAGGC**ATTCATTCATTCATTC**TTTCAGG -3’ |
| Mut-DNMT1(nt-240) | F: 5’- CCTGAAAGTTTGTTTGTTTGTTTGCCTCGG -3’<br>R: 5’- CCGAGGCA**AACAAACAAACAAAC**TTTCAGG -3’ |
| **Primers for ChIP** | |
| DNMT1P(nt -205/-342) | F: 5’- ACGGTTTGCTGGGAGGTG -3’<br>R: 5’- AGGTTGGATTGGAACTGAGG -3’ |

**Supplementary Table S2.** Correlation between DNMT1 expression and clinicopathological characteristics of 149 HCCs.

| | | **DNMT1 expression** | | |
|---|---|---|---|---|
| | **No. of Cases** | **Low expression** | **High expression** | ***P* value** |
| Age (year) | | | | **0.017** |
| ≥ 60 | 34 | 23(67.65 %) | 11(32.35%) | |
| < 60 | 115 | 51(44.35%) | 64(55.65%) | |
| Gender | | | | 0.608 |
| Male | 129 | 63(48.84%) | 66(51.16%) | |
| Female | 20 | 11(55.00%) | 9(45.00%) | |
| Liver cirrhosis | | | | 0.060 |
| Absent | 22 | 15(68.18%) | 7(31.82%) | |
| Present | 127 | 59(46.46%) | 68(53.54%) | |
| HBsAg | | | | 0.198 |
| Negative | 28 | 16(57.14%) | 12(42.86%) | |
| Positive | 121 | 58(47.93%) | 63(52.07%) | |
| ALT(U/I) | | | | 0.566 |
| ≤40 | 75 | 39(52.00%) | 36(48.00%) | |
| >40 | 74 | 35(47.30%) | 39(52.70%) | |
| AFP (ng/ml) | | | | **0.011** |
| ≤400 | 75 | 45(60.00%) | 30(40.00%) | |
| >400 | 74 | 29(39.19%) | 45(60.81%) | |
| Tumor size (cm) | | | | 0.148 |
| ≤5 | 50 | 29(58.00%) | 21(42.00%) | |
| >5 | 99 | 45(45.45%) | 54(54.55%) | |
| Tumor number | | | | 0.081 |
| Single | 125 | 66(52.80%) | 59(47.20%) | |
| Multiple | 24 | 8(33.33%) | 16(66.67%) | |
| Tumor encapsulation | | | | 0.104 |
| Complete | 100 | 45(45.00%) | 55(55.00%) | |
| None | 49 | 29(59.18%) | 20(40.82%) | |
| Tumor differentiation | | | | 0.109 |
| I-II | 108 | 58(53.70%) | 50(46.30%) | |
| III-IV | 41 | 16(39.02%) | 25(60.98%) | |
| Venous invasion | | | | **<0.001** |
| Absent | 51 | 37(72.55%) | 14(27.45%) | |
| Present | 98 | 37(37.76%) | 61(62.24%) | |
| BCLC(CNLC) stage | | | | **0.001** |
| 0+A(Ia+Ib) | 68 | 44(64.71%) | 24(35.29%) | |
| B+C(IIa+IIb+IIIa) | 81 | 30(37.04%) | 51(62.96%) | |

**Note:** Bold values indicate $P < 0.05$, $P$ values from student's $t$-test for age, Fisher's exact test for dichotomous variables, or chi-square when taking all categories into account. HBsAg, hepatitis B surface antigen, ALT, alanine aminotransferase, AFP, alpha-fetoprotein, BCLC, the Barcelona Clinic Liver Cancer staging, CNLC, the Chinese Liver Cancer staging [1].

**Supplementary Table S3.** Univariate cox regression analyses of overall survival (OS) and recurrence free survival (RFS) after surgery in 149 HCCs.

| **Characteristics** | **OS** | | **RFS** | |
|---|---|---|---|---|
| | **HR (95% CI)** | ***P*** | **HR (95% CI)** | ***P*** |
| Age | 0.888(0.546-1.445) | 0.633 | 0.969(0.615-1.528) | 0.892 |
| Gender (male *vs* female) | 0.960(0.534-1.726) | 0.892 | 0.861(0.506-1.465) | 0.581 |
| Liver cirrhosis (present *vs* absent) | 1.378(0.752-2.528) | 0.300 | 0.684(0.383-1.224) | 0.201 |
| HBsAg (positive *vs* negative) | 1.260(0.735-2.159) | 0.401 | 0.828(0.499-1.375) | 0.466 |
| ALT, U/I (> 40 *vs* ≤ 40) | 1.430(0.951-2.151) | 0.086 | 0.721(0.492-1.058) | 0.095 |
| AFP, ng/ml (> 400 *vs* ≤ 400) | 0.296(0.192-0.457) | **<0.001** | 0.463(0.312-0.687) | **<0.001** |
| Tumor encapsulation (none vs complete) | 2.228 (1.368-3.627) | **0.001** | 0.592(0.387-0.905) | **0.016** |
| Tumor differentiation (III-IV vs I-II) | 1.118(0.711-1.759) | 0.629 | 1.384(0.917-2.090) | 0.122 |
| Tumor size, cm (>5 vs ≤5) | 3.238(1.928-5.438) | **<0.001** | 0.422(0.272-0.656) | **<0.001** |
| Tumor number (multiple vs single) | 0.749(0.416-1.346) | 0.334 | 0.929(0.560-1.543) | 0.777 |
| Vascular invasion (present *vs* absent) | 4.133 (2.403-7.109) | **<0.001** | 0.346(0.220-0.546) | **<0.001** |
| BCLC (B+C *vs* 0+A) | 2.929(1.883-4.554) | **<0.001** | 0.397(0.265-0.595) | **<0.001** |
| DNMT1 (high *vs* low) | 0.523(0.345-0.792) | **0.002** | 0.732(0.499-1.073) | 0.110 |

**Note:** Bold values indicate $P < 0.05$. $P$ values from cox regression analysis. CI, confidence interval; HR, hazard ratio.

**Supplementary Table S4.** Multivariate cox regression analyses of OS and RFS after surgery in 149 HCCs.

| Characteristics | OS | | RFS | |
|---|---|---|---|---|
| | HR (95% CI) | *P* | HR (95% CI) | *P* |
| AFP, ng/ml (> 400 *vs* ≤ 400) | 0.470(0.295-0.748) | **0.001** | 0.653(0.426-1.001) | 0.051 |
| Tumor encapsulation (none *vs* complete) | 0.719(0.429-1.202) | 0.208 | 0.859(0.545-1.355) | 0.515 |
| Tumor size, cm (>5 *vs* ≤5) | 0.492(0.282-0.860) | **0.013** | 0.591(0.362-0.963) | **0.035** |
| Vascular invasion (present *vs* absent) | 0.485(0.240-0.979) | **0.043** | 0.527(0.289-0.964) | **0.038** |
| BCLC (B+C *vs* 0+A) | 0.951(0.538-1.679) | 0.862 | 0.846(0.483-1.482) | 0.559 |
| DNMT1 (high *vs* low) | 0.819(0.533-1.260) | 0.365 | 1.109(0.740-1.644) | 0.616 |

**Note:** Bold values indicate $P < 0.05$. *P* values from cox regression analysis. CI, confidence interval; HR, hazard ratio.

**Supplementary Table S5.** KEGG analysis of DMS enriched in PI3K- AKT pathway in Hep3B and PLC/PRF/5 cells with ZNF191stable knockdown.

| Cell line | Hits | Enrichment score | Gene Symbols | *P* value |
|---|---|---|---|---|
| Hep3B | 23 | 1.7357 | ITGA8;INSR;PPP2R2A;LAMC1; MTCP1;LPAR4;PDGFRB;EIF4B; MET;KRAS;COL4A5;IGF2;CCND1; HGF;IGF1R;CDK6;COL4A6;VEGFC; LAMA2;PDGFC;BDNF;COL4A4;TNXB | **0.0067** |
| PLC/PRF/5 | 57 | 1.5156 | PPP2R2C;COL4A1;LPAR5;GHR; EFNA5;PPP2R3C;PHLPP1;PTK2; ITGAV;EPHA2;FGF18;EIF4E; PIK3CA;COL9A3;ITGB8;IRS1; ERBB4;FGFR1;COL9A2;ITGB5; PPP2R2B;CCNE2;PDGFD;FLT1; NTRK2;PIK3R5;ITGA1;SGK1; ITGA5;ITGB6;RXRA;LPAR6; YWHAE;COL6A3;FGF21; GNG11;COL4A5;LAMB1; HGF;LAMA4;VWF;IGF1R; PPP2R2D;COL4A6;VEGFC; JAK3;PDGFC;C8orf44SGK3; BDNF;CREB5;MAGI2;TNXB; FGFR2;IL7;SGK3;ATF6B;KITLG | **0.00077** |

**Supplementary Table S6.** Correlation between ZNF191 and DNMTs mRNA expression in 967 cancer cell lines in TCGA database (Novartis/Broad, Nature 2012).

| Correlated Gene | Cytoband | Spearman's Correlation | p-Value | q-Value |
|---|---|---|---|---|
| **DNMT1** | 19p13.2 | 0.340 | 1.37e-27 | 1.89e-26 |
| **DNMT3A** | 2p23.3 | 0.230 | 4.11e-13 | 1.88e-12 |
| **DNMT3B** | 20q11.21 | 0.148 | 3.699e-6 | 9.009e-6 |
| **DNMT3L** | 21q22.3 | -0.0439 | 0.172 | 0.215 |

**Supplementary Table S7.** Correlation between ZNF191 and DNMTs mRNA expression in HCC tissues (371 patients / 373 samples) in TCGA database.

| Correlated Gene | Cytoband | Spearman's Correlation | p-Value | q-Value |
|---|---|---|---|---|
| **DNMT1** | 19p13.2 | 0.225 | 1.121e-5 | 5.145e-5 |
| **DNMT3B** | 20q11.21 | 0.142 | 6.141e-3 | 0.0158 |
| **DNMT3A** | 2p23.3 | 0.0718 | 0.167 | 0.260 |
| **DNMT3L** | 21q22.3 | 0.0633 | 0.223 | 0.329 |

**Supplementary Table S8.** MDS of *PTEN* and *MTs* gene in Hep3B or PLC/PRF/5 cells with ZNF191 stable knockdown via methylation array

| Gene symbols | MDS of *PTEN* and *MTs* gene |
|---|---|
| PTEN | cg03784654, cg13021550, cg19358349, cg04810794, cg09544159, cg25461644 |
| MTs | cg26640547, cg26937772, cg11530677, cg05958050, cg00519002, cg22655224, cg23687940, cg16393726 |

**Reference:**


1. Zhou J, Sun HC, Wang Z, et al. Guidelines for Diagnosis and Treatment of Primary Liver Cancer in China (2017 Edition). *Liver cancer.* 2018;7(3):235-260.
