# Supplementary material for: ZNF191 alters DNA methylation and activates the PI3K‐AKT pathway in hepatoma cells via transcriptional regulation of DNMT1
Source: Cancer Med. 2022 Jan 28;11(5):1269–80. doi: 10.1002/cam4.4535 (PMC8894703; doi:10.1002/cam4.4535)
Supplement: Supplementary file 3 — Supplementary Material [file CAM4-11-1269-s003.docx]

**Supplementary Materials and Methods**

**Immunohistochemistry (IHC) staining**

HCC tissues were sectioned as 4μm-thick slices and were dewaxed in xylene and rehydrated using 95% ethanol. The endogenous peroxidase activity was quenched by immersing the slides in a 0.3% H_2_O_2_ solution for 30 min at room temperature. Sections were incubated with primary antibodies including anti-DNMT1 (Cell Signaling, Danvers, MA,USA) and anti-ZNF191 (Abcam, Hongkong, China) overnight at 4 °C. The horseradish peroxidase (HRP)-conjugated secondary antibody was applied at 37 °C for 60min and incubated with diaminobenzidine solution, and nuclei were then counterstained with Harris’ Hematoxylin.

The stained tissue slices were scored by two different pathologists blinded to patients’ clinical characteristics. The intensity of IHC staining was scored as 0 (negative), 1 (weak), 2 (medium), and 3 (strong). The percentage of positive cells in the whole tissue slice was recorded. Intensity score and positive rate score were then multiplied to calculate the overall score.

**Quantitative real-time PCR**

Total RNA was extracted from tissues or cultured cells using Trizol reagent, and was reverse transcribed by using SuperScript II reverse transcriptase (Invitrogen, Carlsbad, CA, USA) according to the manufacturer’s protocol. Quantitative real-time PCR was carried out by using SYBR Green Supermix kit (Takara, Dalian, China) with the ABI 7900HT detection system. The list of primers is provided in Table S1. The specificity of PCR product was confirmed by melting curve analysis and gel electrophoresis. DNMT1 and ZNF191 expression levels were normalized to that of the housekeeping gene beta-2-microglobulin (β2-MG). Each reaction was repeated independently at least three times in triplicate.

**Cell lines**

The human HCC cell line Hep3B and PLC/PRF/5, human liver cell L02, and human embryonic kidney cell HEK-293T were purchased (Institute of Biochemistry and Cell Biology, Chinese Academy of Sciences, Shanghai, China). All the cell lines were identified by short tandem repeat typing and mycoplasma detection kit (Shanghai GeneChem Co, Shanghai, China) was used to exclude mycoplasma contamination. Hep3B, PLC/PRF/5 and HEK-293T and were maintained in DMEM with 10% FBS (Invirogen/Gibco). L02 was cultured in RPMI-1640 (GENOM Inc, Hangzhou, China) containing 10% FBS. Cells were maintained at 37°C in a humidified atmosphere of 5% CO2.

**Vectors and constructions**

The *DNMT1* promoter were generated by PCR and cloned into the luciferase reporter gene vector, pGL3-basic (Promega, Madison, WI, USA). The fidelities of the constructs were confirmed by sequencing. Site-directed mutations were performed using a reverse-PCR and DpnI based kit (TOYOBO, Osaka, Japan). All the primers used in constructing and mutating vectors are listed in Table S1.

Expression vector pCMV-Myc-ZNF191-FU (full length of ZNF191) and pCMV-Myc-ZNF191-NF (Short isoform of ZNF191, encoding 1-189 amino acids, named as ZNF191-NF, without C2H2 zinc finger domain) were constructed as previous reported ^[1](#_ENREF_1)^.

**Lentivirus production and cell transduction**

pLVTHM-shZNF191 vector was constructed as described previously[^2^](#_ENREF_2). NC and target sequences are listed in Supplementary Table S1. The multiplicity of infection (MOI) values in transducting different cell lines are: HEK-293T (MOI = 1); L02 (MOI = 10); Hep3B (MOI = 10); PLC/PRF/5(MOI = 10).

**RNA interference**

The cells were transfected with annealed double-stranded DNMT1 or ZNF191 small interfering RNA (DNMT1-siRNA, ZNF191-siRNA) or a nonspecific scrambled control siRNA (synthesized from Shanghai GenePharma Co, Shanghai, China) at a final concentration of 100nM using LipofectAMINE 2000 reagent (Invitrogen) according to the manufacturer’s instructions. The sequences of small RNAs are listed in Table S1.

**Western blotting**

The total proteins were extracted by using the RIPA lysis buffer (Beyotime, Shanghai, China). Protein samples were separated by SDS-PAGE and then transferred onto nitrocellulose membranes (Millipore, Bedford, MA, USA). After blocking, the membranes were incubated with specific antibodies against different proteins at 4℃ overnight, followed by incubation with horseradish peroxidase-conjugated secondary antibody. Immunoreactivity was visualized by enhanced chemiluminescence (ThermoFisher, Waltham, MA, USA). The related antibodies we used included α-tubulin (Proteinteck, Rosemont, IL, USA), anti-DNMT1 (Abcam, Cambridge, UK), anti-ZNF191(Sigma-Aldrich, St. Louis, MO, USA), anti-AKT and anti-Phospho-AKT(Ser473) (Cell Signaling, Danvers, MA,USA).

**Cell Counting Kit-8 (CCK-8) assay**

Cell proliferation assay was performed using CCK-8 (Dojindo Laboratories, Kumamoto, Japan) according to the manufacturer’s instructions. Briefly, hepatoma cells transfected with DNMT1 siRNA or / and ZNF191 siRNA were seeded into 96-well plates at a density of 10^3^ cells/well with 100 μl culture medium. At 1-7 days after transfection, 10 μl CCK-8 reagent was added to each well. The plates were incubated in dark at 37℃for 2 h, and absorbance at 450 nm wavelength was measured. Independent experiments were repeated for five times.

**Colony formation assay**

Cells (500 -1000 cells /well) were plated in 3.5cm dishes. At day 14, the plates were fixed in 4% paraformaldehyde, stained with 1% crystal violet, and the numbers of colonies were then counted to evaluate cell proliferation. The assay was analyzed in three independent experiments.

**Statistical analysis**

In real-time PCR, relative gene expression levels were calculated as described previously.[^1^](#_ENREF_1) A 2-fold change threshold was set for identifying significant changes in gene expression. The other data have been presented as the mean ± standard deviation (mean ± SD). The significance of the differences was determined by Student *t* test. The statistical significance was set at a *P* value of < 0.05. Statistical analyses were performed with GraphPad Prism 6.0 (GraphPad, San Diego, CA, USA) and the SPSS 12.0 (SPSS Inc, Chicago, IL) statistical software package.

**References**

**1.** Liu G, Jiang S, Wang C, et al. Zinc finger transcription factor 191, directly binding to beta-catenin promoter, promotes cell proliferation of hepatocellular carcinoma. *Hepatology.* Jun 2012;55(6):1830-1839.

**2.** Wu D, Liu G, Liu Y, et al. Zinc finger protein 191 inhibits hepatocellular carcinoma metastasis through discs large 1-mediated yes-associated protein inactivation. *Hepatology.* Oct 2016;64(4):1148-1162.
